# Ultrasound-Responsive Smart Drug Delivery System of Lipid Coated Mesoporous Silica Nanoparticles

**DOI:** 10.3390/pharmaceutics13091396

**Published:** 2021-09-03

**Authors:** Muhammad Umair Amin, Sajid Ali, Imran Tariq, Muhammad Yasir Ali, Shashank Reddy Pinnapreddy, Eduard Preis, Christian Wölk, Richard D. Harvey, Gerd Hause, Jana Brüßler, Udo Bakowsky

**Affiliations:** 1 Department of Pharmaceutics and Biopharmaceutics, University of Marburg, Robert-Koch-Str. 4, 35037 Marburg, Germany; umairami@staff.uni-marburg.de (M.U.A.); sajidalichishti@gmail.com (S.A.); imran.pharmacy@pu.edu.pk (I.T.); m.yasirali14@gmail.com (M.Y.A.); shashank.pinnapireddy@pharmazie.uni-marburg.de (S.R.P.); eduard.preis@pharmazie.uni-marburg.de (E.P.); jana.bruessler@staff.uni-marburg.de (J.B.); 2 Angström Laboratory, Department of Chemistry, Uppsala University, 75123 Uppsala, Sweden; 3 Punjab University College of Pharmacy, University of Punjab, Lahore 54000, Pakistan; 4 Faculty of Pharmaceutical Sciences, GC University Faisalabad, Faisalabad 38000, Pakistan; 5 Institute of Pharmacy, Pharmaceutical Technology, Faculty of Medicine, Leipzig University, Eilenburger Straße 15a, 04317 Leipzig, Germany; christian.woelk@medizin.uni-leipzig.de; 6 Department of Pharmaceutical Chemistry, University of Vienna, Althanstraße 14, A-1090 Vienna, Austria; richard.harvey@univie.ac.at; 7 Biocenter, Martin Luther University Halle-Wittenberg, Weinbergweg 22, 06120 Halle, Germany; gerd.hause@biozentrum.uni-halle.de

**Keywords:** mesoporous silica nanoparticles, lipid coating, ultrasound triggered release, mechanical index, cellular uptake

## Abstract

The immediate release of chemotherapeutics at the target site, along with no premature release in circulation is always challenging. The purpose of this study was to develop a stimuli responsive drug delivery system, composed of lipid supported mesoporous silica nanoparticles (MSNPs) for triggered drug release at the target site and simultaneously avoiding the premature release. MSNPs with a higher drug loading capacity and very slow release were designed so as to enhance release by FDA approved US-irradiation. Doxorubicin, as a model drug, and perfluoropentane (PFP) as a US responsive material, were entrapped in the porous structure of MSNPs. Lipid coating enhanced the cellular uptake and in addition provided a gatekeeping effect at the pore opening to reduce premature release. The mechanical and thermal effects of US induced the conversion of liquid PFP to a gaseous form that was able to rupture the lipid layer, resulting in triggered drug release. The prolonged stability profile and non-toxic behavior made them suitable candidate for the delivery of anticancer drugs. This smart system, with the abilities of better cellular uptake and higher cytotoxic effects on US-irradiation, would be a good addition to the applied side of chemotherapeutic advanced drug delivery systems.

## 1. Introduction

Recent developments in nanostructures, such as polymeric nanoparticles; liposomes; dendrimers; micelles; and inorganic nanocarriers including gold, quantum dots, iron oxide, and mesoporous silica nanoparticles have become attractive and advantageous systems for drug delivery and biomedical imaging [1]. Smart drug delivery systems are very attractive due to being stimuli responsive, where release of the drug is triggered by physical, mechanical, or chemical means [2,3]. Unfortunately, the treatment of cancer is still elusive and accompanied by certain side effects and drug resistance. The main challenge related to chemotherapeutics is the distribution of drugs in nonspecific healthy tissues, leading to undesired effects [4]. One of the major problems of the conventional dosage forms of chemotherapeutics is the premature release of the drug before reaching the target site. As a result, a small quantity of chemotherapeutics reaches the target tumor, leading to ineffective results [5]. Although this issue has been addressed by recent developments in stimuli responsive drug delivery in a controlled manner, these stimuli responsive systems still need to overcome many challenges in their application from bench to bedside [6,7].

In nanoscale dosage forms, the release of drugs from nanoparticles depends upon the degradation of the system. To overcome this limitation, the development of stimuli responsive drug delivery systems has been a major area of interest for the last few years. These stimuli responsive systems are categorized into endogenous stimuli, including pH; enzymatic cleavage; oxidative-reductive reactions; and exogenous stimuli, including temperature, ultrasound (US), light, magnetic, and electrical fields [8,9,10]. From exogenous stimuli, ultrasound offers many advantages due to its non-ionizing features, cost effectiveness, and noninvasive nature. In addition, site-specific outcomes with minimum side effects can be obtained due to its deep penetration, depending upon exposure and frequency [11,12]. Different ultrasound intensities have been utilized, not only to trigger release from the drug carrier, but also for their tracking in the body [13]. Evidently, with Mechanical Index (MI) < 0.5, US possesses lower acoustic energy with lesser bursting effects of contrasting agents, and ultimately results in prolonged imaging. In contrast MI > 0.5 can cause the destruction of perfluoropentane (PFP), an ultrasound contrasting agent. An MI of 1.4 has already been applied for instantaneous bursting of perfluorocarbons [14]. For diagnostic purpose, destruction of the contrasting agent is considered as a limiting factor, but it can be potentially utilized to trigger the release of the drug at the target site. The drug release from the carrier is due to the thermal and mechanical effects of US [15]. The aim of this study was to develop an ultrasound responsive drug delivery system that can release the payload on demand at the tumor sight without any harmful effects to healthy tissues. Although low frequency and high intensity focused ultrasound (HIFU) radiation has been used in drug delivery systems, however due to higher risks of thermal and mechanical injuries, their applications are limited [16,17,18]. Herein, FDA approved US specifications have been utilized for triggered drug release.

Compared to conventional drug delivery systems, nanocarriers are of utmost importance because of their better drug delivery, decreased side effects, and easy modification of physicochemical characteristics [19]. The development of inorganic and biocompatible nano-systems, such as porous silica, have shown better therapeutic effects in gene- and chemotherapy compared to other nanocarriers [20]. Various features such as larger surface area (>700 m^2^g^−1^), mechanical strength, tailorable pore size (2–10 nm) and pore volume (>1 cm^3^g^−1^), hydrophilicity and hydrophobicity, high drug loading capacity and sustained release, internal and external surface modification, and dual surface for drug attachment make them more suitable for a wide range of applications for medical and diagnostic purposes [21,22].

Liquid filling to the porous structure of MSNPs by capillary action and its conversion to bubbles is a suitable approach for the entrapment of low boiling point materials. As an established ultrasound contrasting agent with a lower boiling point of 28 °C, PFP is an ideal candidate for loading into the porous structure. PFP is hydrophobic in nature and, therefore, in the blood it remains undissolved. Consequently, it is useful for stabilizing the nanobubbles in US diagnostics, and with this tendency, it ultimately exhibits a longer lifespan on hydrophobic surfaces. In comparison to lipid entrapped microbubbles, MSNPs can entrap and hold the interfacial liquid in their pores for a longer duration due to their larger surface area and hydrophobicity [23]. The entrapped liquid PFP in the porous structure can be converted to vapors due to its low boiling point to produce a larger volume of gas inside the pores. Advancements in sonochemistry has revealed that ultrasound waves have some physical and chemical effects. Physical effects are exerted by thermal and mechanical induction, while chemical effects are produced by chemical reactions. Induction of the mechanical and/or thermal effects of US can be utilized to convert lower boiling point liquids to gas, exerting pressure to the carrier for the triggered release of the drug [24,25].

Liposomes are similar to cell membrane and always remain in focus due to higher biocompatibility, faster degradability, and lower immunogenicity. Therefore, they are used as drug carriers for better cellular uptake of anticancer drugs and delivery of genetic materials [26,27]. These distinguishing features of liposomes, in combination with MSNPs, can enhance biocompatibility and result in improved cellular uptake. The problems related to liposomes, including leakage and stability in circulation, has already been reported; however, the incorporation of cholesterol to increase cohesiveness can minimize the leakage problem [28]. The biocompatibility of MSNPs can be enhanced by lipid coating to produce very helpful and synergetic effects. The enhanced effects of lipid coated MSNPs compared to non-lipid coated MSNPs have already been reported [29,30].

**Scheme 1:**
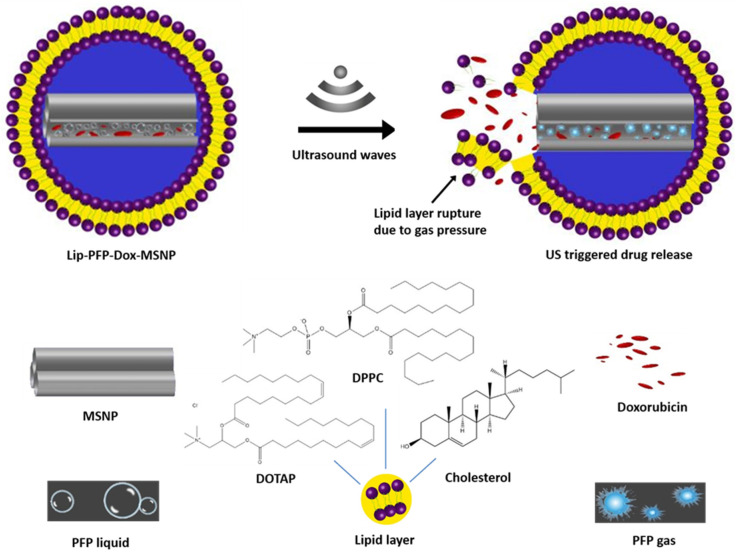
Schematic illustration of US-responsive drug delivery system based on lipid coated MSNPs loaded with Dox and PFP.

Lipid coating, as a gatekeeper at the pore openings of MSNPs, provides an intact barrier against the premature release of the drug and liquid inside the pores under inert conditions [31], but the application of US waves, with their thermal effects to thermos-sensitive liposomes, can result in a loss of gate keeping at the MSNP pore openings. Therefore, the combined energy of the mechanical and thermal effects on the vaporization of the liquid inside the pores and rupturing of the lipid coating enhance the release of the drug [32]. Herein, we have developed a US-responsive drug delivery system, as presented in Scheme 1, which has shown stability for a longer duration and can trigger the release of the drug on exposure to US-irradiation.

## 2. Materials and Methods

### 2.1. Materials

Tetraethylorthosilicate (TEOS, ≥99%) and dimethyl sulfoxide (DMSO, ≥99.5%) were purchased from Merck KGaA (Darmstadt, Germany). Cetyltrimethylammoniumbromide (CTAB, >99%) and sodium hydroxide (NaOH, >99%) were purchased from Carl Roth GmbH & Co. KG (Karlsruhe, Germany). Fluorescein-5 isothiocyanate (FITC, 90% HPLC) and 3-aminopropyl triethoxysilane (APTES, 99%) were purchased from Sigma–Aldrich Chemie (Taufkirchen, Germany). 1,2-dipalmitoyl-sn-glycero-3-phosphocholine (DPPC), Cholesterol, and 1,2-dioleoyl-3-trimethylammonium-propane (DOTAP) were gifted from Lipoid GmbH (Ludwigshafen, Germany). Doxorubicin hydrochloride (Dox, >95%) was purchased from Fluorochem Ltd. (Glossop, UK). Dulbecco’s Modified Eagle’s (DMEM) Medium and fetal calf serum were purchased from Biochrom GmbH (Berlin, Germany) and PAA Laboratories GmbH (Cölbe, Germany), respectively. 4′,6-diamidino-2-phenylindole (DAPI, ≥98%), 3-(4,5-dimethylthiazol-2-yl)-2-5-diphenyltetrazolium bromide (MTT) dye, and uranyl acetate were purchased from Sigma–Aldrich Chemie (Germany). Perfluoro-n-pentane (PFP, 90%) was purchased from abcr GmbH (Karlsruhe, Germany).

### 2.2. Preparation of Lip-PFP-Dox-MSNPs

#### 2.2.1. Preparation of Liposomes

Liposomes were prepared by a slight modification of the thin layer hydration method [33]. The molar mass ratio of DPPC, Cholesterol, and DOTAP was 85:12:3 in the final liposomal formulation. Briefly, the desired amount of lipids in chloroform:methanol (2:1 *v*/*v*) was added to a round-bottomed flask and further diluted. The organic solvents were evaporated at 45 °C under vacuum pressure with a rotary evaporator Heidolph Laborota 4000 efficient (Heidolph Instruments, Schwabach, Germany). The obtained thin lipid layer was completely hydrated with PBS (pH 7.4) buffer to obtain a final concentration of 10 mg/mL lipid. The mixture was subjected to sonication in an ultrasound bath for 15 min at 45 °C. Finally, the lipid mixture was extruded through a 100 nm polycarbonate filter at transition temperature to attain uniformed size distribution [34].

#### 2.2.2. Fabrication of MSNPs

Mesoporous silica nanoparticles (MSNPs) were fabricated by hydrolysis and condensation of TEOS, a two-step method. MSNPs preparation was carried out by the already reported method, with little modification [35]. Forty-eight milliliters of ultrapure water was mixed with 350 µL of 2 M NaOH and 200 mg of CTAB surfactant. The mixture was stirred at 80 °C for 2 h at 350 rpm to obtain pellucid solution in the form of packed micelles. Afterwards, 400 µL of TEOS was added in the form of drops, and the mixture was stirred overnight with the same speed and temperature. The final molar mass ratios of CTAB:TEOS:NaOH:H_2_O were 0.55:1.8:0.7:2667, respectively. Finally, a milky solution was obtained and subjected to centrifugation at 16,000× *g* for 20 min, and surf-MSNPs were collected as pellets. For the surfactant removal, particles were extracted in an oil bath with ethanol:HCl (19:1) at 80 °C. MSNPs were further washed twice with ethanol and water with the above mentioned centrifugal force and time. After complete surfactant removal, pure MSNPs were resuspended in water, lyophilized, and stored at −20 °C for further use.

#### 2.2.3. Preparation of Dox-MSNPs

An amount of 2.5 mg of dried MSNPs was suspended in 3 mL of deionized water and sonicated in a sonication bath for 5 min to break the aggregates of the particles. Three milliliters of Dox solution in deionized water (2 mg/mL) was added to the abovementioned MSNPs suspension, and the final mixture was stirred overnight at 350 rpm to attain equilibrium. The mixture vial was covered with aluminum foil for light protection. After overnight stirring, the mixture was centrifuged at 16,000× *g* for 30 min and the supernatant was collected. The settled drug-loaded particles were dispersed in deionized water and the process was repeated three times to remove the free Dox, and each time supernatant was collected. Finally, Dox-MSNPs were suspended in deionized water for lyophilization. The supernatant was measured for Dox concentration at 495 nm with a Shimadzo UV-Visible spectrophotometer UVmini-1240 (Shimadzo Corp, Kyoto, Japan). The amount of Dox entrapped in the MSNPs was then measured accordingly [36].

#### 2.2.4. Preparation of PFP-Dox-MSNPs and Lipid Coating

Dox-MSNPs, corresponding to 2.5 mg MSNPs, were mixed with 200 µL of perfluoropentane (PFP). To avoid evaporation of PFP, sonication was carried out for 5 min at 4 °C. Ultrasound contrasting agents can penetrate to the pores of MSNPs through capillary action. After further incubation of particles for 24 h at 4 °C, extra PFP was evaporated at room temperature, and PFP-Dox-MSNPs were obtained in a dried form.

Liposomal coating to MSNPs was carried out using previously optimized MSNPs to a liposomal mass ratio of 1:0.72. The definite amount of liposomes was added to PFP-Dox-MSNPs and pipetted vigorously to form the lipid coating on the particle surface to obtain Lip-PFP-Dox-MSNPs [37]. As a control, Lip-Dox-MSNPs without the addition of PFP were also prepared.

### 2.3. Size and Surface Charge Measurement

Dynamic light scattering was performed for the size measurement of MSNPs, liposomes, and lipid coated MSNPs by Zetasizer NanoZS (Malvern Panalytical, Kassel, Germany). A HeNe laser at a wavelength of 633 nm was utilized with a light scattering angle of 173°. For surface charge measurement, Laser Doppler Velocity (LDV) was used with scattered light at an angle of 17°. Three independent MSNPs and liposomal formulations were measured. Each measurement comprised 10 runs, and each formulation was measured in triplicate. The intensity distributions of three independent formulations are presented as averages with standard deviations. Likewise, three formulations were measured for polydispersibility index (PDI) to acquire the average with standard deviation.

### 2.4. Morphological Studies

The particle size distribution of MSNPs was initially visualized with Atomic Force Microscopy (AFM). Briefly, a diluted sample was placed on a silica surface that was fixed on a glass slide. After removal of extra liquid, the samples were observed with a Nanowizard 3 Nanoscience (JPK Instruments, Berlin, Germany) by using HQ:NSC14/AL_BS cantilever tips. Random images were obtained in amplitude trace and measured height trace mode, and results are presented in the Supplementary Materials (Figure S1).

The TEM micrographs were acquired at 300 kV accelerated voltage with the help of a transmission electron microscope (TEM JEOL 3010, 300 kV) at Philipps University, Marburg. Samples were placed on copper grids and observed under microscope. The samples were coated with a very thin layer of carbon due to the non-conductive behavior of the particles. Lipid coated MSNPs were negatively stained at a dilution of 1:1 *v*/*v* with 2% uranyl acetate by incubating for 30 min, and samples were observed at the above mentioned specifications. Another set of US-irradiated lipid coated MSNPs was observed at an accelerated voltage of 80 kV with EM900 TEM (Carl Zeiss Microscopy GmbH, Jena, Germany) at the MLU Halle Wittenberg.

For CryoTEM micrographs, vitrified samples were prepared with a blotting procedure under a controlled temperature and humidity in a chamber using an EM GP grid plunger (Leica, Wetzlar, Germany), and micrographs were obtained at MLU Halle Wittenberg. An EM grid coated with holey carbon film (Cflat, Protochips Inc., Raleigh, NC, USA) was used for the sample preparation; 6 µL of the sample was placed on the grid, and any excess was removed with a filter paper. After vitrification of the grid by rapid plunging into liquid ethane (above its freezing point), the specimen was stored below 108 K and then transferred to a microscope for investigations. Micrographs were obtained at 120 kV voltage with a Libra 120 Plus transmission electron microscope (Carl Zeiss Microscopy GmbH, Germany) equipped with a Gatan 626 cryotransfer system and BM-2K-120 dual-speed on-axis SSCCD camera (TRS).

### 2.5. Stability Studies of Lip-PFP-Dox-MSNPs

**Figure 1:**
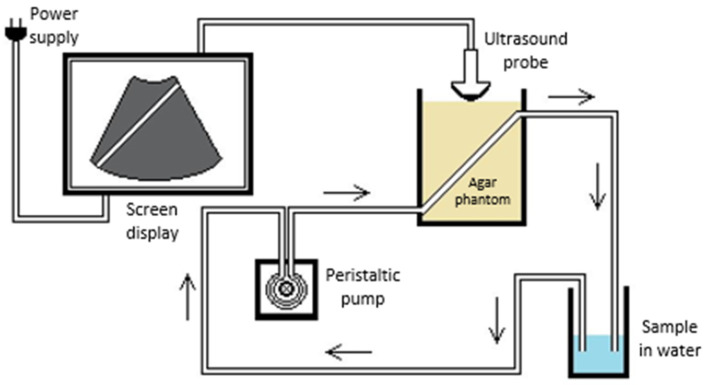
Experimental agar phantom model for US contrast evaluation of PFP loaded drug carrier.

The US contrast studies were performed with specifications approved by the FDA. A commercially available device, eZono 3000 (eZono AG, Jena, Germany), designed for clinical purposes, fitted with a probe with a maximum intensity of 5.17 mW/cm^2^, MI 0.7, and a center frequency of 3.5 MHz, was utilized for ultrasound characterizations. To imitate the blood vessels and soft tissues, a self-designed agar phantom model was used with a set of tubes. The system was further equipped with a peristaltic pump to mimic the fluid circulation in a continuous but constant manner in one direction. The US contrast could easilly be observed on the screen. A complete layout of the system is presented in Figure 1.

To observe the contrast in dynamic conditions, 100 µL of the PFP loaded MSNPs sample was added to 100 mL of the PBS buffer circulating at 37 °C. The US contrast stability due to PFP entrapment was evaluated for a time span of 120 h by adding 100 µL of the sample at different time intervals.

In addition to US contrast stability, premature drug release studies were performed to evaluate the effect of PFP (into the pores) on lipid layer integrity and MSNP drug retention capacity under inert conditions. As it was assumed that the release of the drug would only be triggered by US-irradiation due to the conversion of liquid PFP to a gaseous form, it was mandatory to evaluate the drug release in the absence of US-irradiation as far as the stability of the carriers was concerned. Premature drug release studies were performed for 10 days by incubating Dox-MSNPs, Lip-Dox-MSNPs, and Lip-PFP-Dox-MSNPs in PBS (pH 7.4) under static conditions at 4 °C. At different time intervals, samples were collected, and the amount of drug release was evaluated with a UV-visible spectrophotometer.

### 2.6. Ultrasound Triggered Drug Release

For in vitro drug release studies, another self-designed agar phantom model was used, with a central cavity of 15 mL. The cavity was filled with PBS buffer (pH 7.4), and stirring speed was set to 150 rpm. A definite amount of Lip-PFP-Dox-MSNPs was added to the buffer and subjected to US-irradiation with a probe at a maximum intensity of 351.37 mW/cm^2^, MI 1.4, and a center frequency of 12 MHz. Samples were collected at predefined time intervals and replaced with fresh medium. Samples were subjected to centrifugation, and supernatants were measured UV-Vis spectrophotometrically for the amount of drug release. To evaluate the US triggered release effects, a comparative study was made by adopting same procedure in the absence of US.

### 2.7. Measurement of Gas Produced by Vaporization

The presence of liquid PFP inside pores, and its conversion to gas upon US-irradiation, was evaluated with a two-way Luer lock stopcock model consisting of a connected double syringe system [38]. In short, 2 mL of Lip-PFP-Dox-MSNPs were diluted with purified water to achieve a concentration of 2.5 mg/mL in a Hamilton gas-tight 10 mL syringe. This was further connected to a two-way Luer lock stopcock, the plunger was depressed to completely remove the air, and the stopcock was closed. To avoid any air pocket, a 250 µL syringe containing 20 µL of water was connected to the other side of the lock. Afterwards, a vacuum was created by withdrawing the plunger of the larger syringe to two to three times the volume of the dispersion. The plunger was released after holding it in the same position for 2 min. Finally, the amount of air produced was measured by the air pocket between the dispersion and the plunger of the larger syringe (Supplementary Materials, Figure S4).

### 2.8. Cell Culture Experiments

MDA-MB 231 (ATTC, Manassas, VA, USA), epithelial adenocarcinoma breast cancer cells, were used for cell culture experiments. The cells were cultivated with high glucose Dulbecco’s Modified Eagle’s Medium (DMEM) with 10% fetal calf serum. The cells were provided with suitable humidity, along with 7% CO_2_ at 37 °C for optimal growing conditions.

#### 2.8.1. In Vitro Cytotoxicity (MTT Assay)

To evaluate the cytotoxic effects and drug delivery, cells were seeded at a density of 40 × 10^4^ cells/cm^2^ in a petri dish (20 cm^2^) in 5 mL of the medium and incubated in 7% CO_2_ atmosphere at 37 °C for 24 h. Afterwards, the medium was replaced with fresh medium containing Lip-Dox-MSNPs, Lip-Dox-MSNPs (US), Lip-PFP-Dox-MSNPs, and Lip-PFP-Dox-MSNPs (US) with different Dox concentrations (ranging from 200 µg/mL to 1.56 µg/mL) and further incubated for 4 h. Free Dox in the same concentrations was used as standard. Following this, the medium was aspirated and fresh medium was added to irradiate the sample with US for 5 min. Uniform US irradiation was attained by changing the position of the petri dish, followed by overnight incubation. On the following day, the old medium was replaced with fresh medium containing 0.6 µg/mL of 3-(4,5-dimethylthiazol-2-yl)-2,5-diphenyltetrazolium bromide (MTT), which was incubated for a further 4 h. After aspiration of the medium, DMSO was added and the petri dishes were stirred to dissolve formazan crystals. The solution was then transferred to a 96-well plate to be measured for absorbance at 570 nm with a microplate reader (FLUOstar, BMG Labtech GmbH, Ortenberg, Germany). The measurements were carried out in triplicate and results were represented as average. The following formula was used to calculate the cell viability:

(1) $$\%\;\mathrm{Viabilty}=\frac{\left(Ab_{\mathrm{Sample}}\;–\;Ab_{\mathrm{Blank}}\right)}{\left(Ab_{\mathrm{Control}}\;–\;Ab_{\mathrm{Blank}}\right)}\times 100\;\%$$

where *A*b_Blank_ is the absorbance of no cells, while *A*b_Sample_ and *A*b_Control_ are the treated and non-treated cells, respectively. The whole of the experiment was performed in triplicate, and results were calculated as an average along with standard deviation.

#### 2.8.2. Cellular Uptake Studies

For tracking of the cellular uptake of the carrier, MSNPs were labeled with fluorescein-5 isothiocyanate (FITC) by slight modification of the already reported method [39]. Briefly, 1.1 mg of FITC was mixed into 600 µL of ethanol and 2.5 µL of 3-aminopropyl triethoxysilane (APTES), and the solution was stirred under nitrogen atmosphere for 2 h. Then, TEOS was mixed with the FITC-APTES solution before adding to the CTAB solution, and the molar mass ratios were kept the same as for the non-labelled MSNPs preparation. The cells were seeded at a cell density of 40 × 10^4^ cells/cm^2^ with DMEM medium in a petri dish (20 cm^2^) and incubated for 24 h. Following this, cells were thoroughly washed with PBS buffer and further incubated with definite concentrations of Dox, Lip-Dox-MSPs, and Lip-PFP-Dox-MSNPs in fresh medium for 4 h. The medium was replaced and the petri dish, treated with Lip-PFP-Dox-MSNPs, was exposed to US-irradiation for 5 min. Later on, cells were washed with PBS and fixed with 4% paraformaldehyde by incubating with 300 µL for 10 min. Cells were further washed with PBS buffer, and nuclei were stained with 300 µL of 4′,6-diamidino-2-phenylindole (DAPI) for 25 min incubation. After washing the cells, the cover slip was placed on a glass slide and observed under confocal laser scanning microscope (Zeiss, LSM 510, Jena, Germany). A UV laser of 364 nm was used for excitation of DAPI, and emissions were detected at 385–470 nm. Similarly, FITC was excited at 495 nm and Dox was excited at 480 nm. The emission of FITC and Dox were detected at 519 and 560–590, respectively.

## 3. Results and Discussion

### 3.1. Synthesis of MSNPs, Lip-MSNPs; Optimization and Characterization

**Figure 2:**
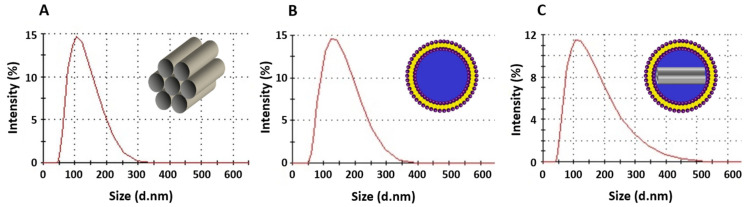
DLS measurements for size distribution of (**A**) MSNPs, (**B**) Liposomes, and (**C**) Lip-MSNPs.

**Figure 3:**
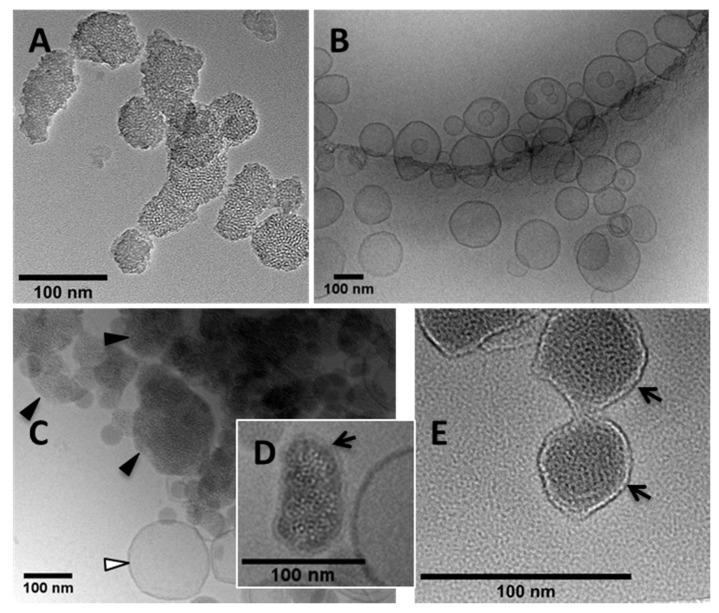
TEM micrographs of various formulations. (**A**) MSNPs showing mesoporous structure. (**B**) CryoTEM micrograph of cationic liposomes. (**C**,**D**) CryoTEM micrograph of lipid coated MSNPs. (**C**) Black triangles pointing to the mesoporous structure of lipid coated MSNPs, and white triangles pointing to liposome. (**D**) Black arrow indicates a membrane-like structure around lipid coated MSNPs. (**E**) Negatively stained lipid coated MSNPs. Black arrow indicates a membrane-like structure around lipid coated MSNPs.

Cetyltrimethylammonium bromide (CTAB) was used as an organic template to impart a porous character by forming packed micelles in a basic condition. Mesoporous silica nanoparticles (MSNPs) were successfully prepared by the slight modification of a previously reported method, where a silica source, tetraethylorthosilicate (TEOS), was coated onto the surface of a CTAB-template. MSNPs were initially characterized by Dynamic Light Scattering (DLS) for size distribution and for surface charge, where the average particle size was 118 ± 2 nm with PDI 0.159, indicating a uniform dispersion with broad size distribution (Table 1). The size distribution curve is shown in Figure 2A, demonstrating particle sizes between 50 nm and 300 nm, with a mode at around 100 nm. By washing the MSNPs, the surfactant was removed from the pores, and this was confirmed by the shift of surface charge from +34 ± 5 mV to −17 ± 4 mV by Laser Doppler Velocimetry (LDV). Size distribution of the MSNPs, along with PDI and zeta potential, are shown in Table 1 and Figure 2. TEM images clearly show the mesoporous structure of the MSNPs (Figure 3A).

Liposomes were prepared by thin layer hydration method according to the protocol developed by our research group. Initially, different molar ratios of various lipids were used, and finally a 85:3:12 molar mass ratio of DPPC, DOTAP, and cholesterol were selected for MSNPs coating due to their relatively lower toxicity. Cationic liposomes act as counter charge moieties to the anionic surfaces of MSNPs and form a uniform coating. Furthermore, liposomal surface tension also plays a critical role in the lipid coating of MSNPs. The major reason behind lipid coating is to avoid premature drug leakage due to the gatekeeping effects, and it simultaneously sustains the release of the drug [40,41]. Above all, liposomes exhibit higher biocompatibility and lower toxicity due to their resemblance to cell membrane, which results in enhanced cellular uptake [29,42]. According to the DLS of liposomes prepared by the above mentioned method, the size and PDI were 126 ± 12 nm and 0.177, respectively. The surface charge measured by LDV was +18 ± 4 mV, as shown in Table 1. The distribution curve of their size is presented in Figure 2B. The size of the liposomes was further confirmed by CryoTEM, where they were found to be in the range of 100 nm, and the results are shown in Figure 3B. The images further confirm the unilamelar character of the vesicles, an effect of the positive charge; consequently, a coating of MSNPs with a single lipid bilayer can be expected rather than a coating of multiple bilayers.

The uniform lipid coating to MSNPs was optimized by using different MSNPs to liposomes mass ratios. MSNPs are solid and rigid in nature; therefore, they can be easily entrapped in the softer liposomes through applied shear by vortex mixing and pipetting. Nevertheless, CryoTEM images demonstrate that residual empty cationic liposomes are present in the sample (Figure 3C, white triangle), next to the lipid coated MSNPs (Figure 3C, black triangles).

According to DLS measurements, after lipid coating to MSNPs, the size has been increased by 12 nm, and the final size of the lipid coated MSNPs was 130 ± 10 nm. The lipid coating was further indicated by a shifting of the negative charge of the MSNPs to positive due to cationic liposomes. As shown in Table 1, the charge was shifted from −17 ± 4 mV to +8 ± 3 mV after lipid coating. Surface area and pore size were evaluated by nitrogen adsorption–desorption isotherm, and the results are presented in the Supplementary Materials (Figure S2). The surface area of MSNPs, measured by Brunauer–Emmett–Teller (BET) was 1054 m^2^/g, and the pore diameter evaluated by Barrett–Joyner–Halenda (BJH) was around 2.43 nm. The hysteresis loop in the adsorption–desorption isotherm (Figure 2A) and pore size confirmed the mesoporous nature of the particles. Visualization of MSNPs with AFM from different areas has shown that the particle size is consistent with DLS results, and there are no aggregates. The AFM images in amplitude trace and measured height trace, along with graphs, can be observed in the Supplementary Materials (Figure S1). A detailed evaluation (higher magnification) of the TEM images (CryoTEM and negative staining) show a single thin layer around the Lip-MSNPs (Figure 3D,E, black arrows), which must be a lipid bilayer. Consequently, the liposomes seem to coat the surface of the MSNPs rather than include them in the aqueous pore, a process that can be explained by electrostatic attraction.

### 3.2. Drug Loading and Preparation of Lip-PFP-Dox-MSNPs

The drug loading capacity of MSNPs was investigated using Dox, a cytostatic drug, as a tumor therapeutic agent. Six milligrams of Dox was mixed with 2.5 mg of MSNPs, as mentioned in the experimental section, and the calculated percentage drug entrapment was 48.89 ± 5.16%. A higher amount of the drug can be entrapped due to the larger surface area of MSNPs [2,43,44] and resulted in 53.95% drug loaded contents. The electrostatic interaction between the negatively charged MSNPs and the positively charged amine group in Dox enhance the drug entrapment [45]. The higher drug loading capacity is related to the availability of a large surface area of porous structure. US active PFP was incorporated into lyophilized Dox-MSNPs by sonication with 100% power at 37 kHz for 10 min. Capillary filling of PFP liquid to the pores of MSNPs has already been reported in the literature [46]. The selection of US contrasting agent PFP was based on its low boiling point (28 °C). The incubation of Dox-MSNPs with PFP at room temperature resulted in extra liquid PFP evaporation. At the same time, the liquid PFP in the porous structure was retained by the capillary force of the pores, which increased the boiling point of the liquid. However, the combined effect of US and body temperature (37 °C) dislodge and vaporize the liquid from the pores, and the produced bubbles were able to show US contrast. Post-loading of PFP to Dox-MSNPs was advantageous in two ways. Firstly, functionalization and grafting of MSNPs reduced the drug loading surface area of MSNPs. Secondly, it was observed that the pre-loading of PFP to MSNPs resulted in PFP evaporation during Dox loading and washing. Such formulations have shown vey negligible and poor US contrast. Therefore, dried Dox-MSNPs were used to incubate with PFP for efficient loading and longer retention. After evaporating extra liquid PFP, the dried PFP-Dox-MSNPs were immediately coated with a lipid layer. For lipid coating, the same optimized ratio of lipid to MSNPs was used. The intact lipid layer on the surface of MSNPs acted as a barrier and kept the PFP inside the pores of the MSNPs. For further experiments, particles were resuspended in water.

### 3.3. Ultrasound Contrast Characterization and Stability of Carrier

**Figure 4:**
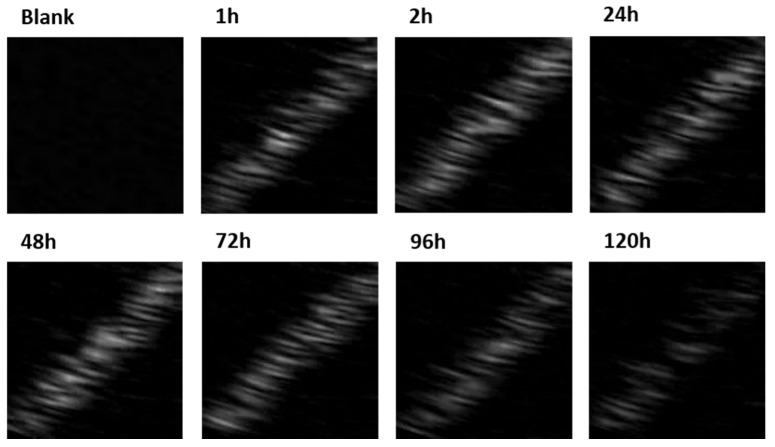
Representative images US contrast of PFP loaded Lip-MSNPs at different time intervals with 3.5 MHz and MI 0.7.

The US contrast measurements were first optimized with Lip-PFP-MSNPs without drug loading, and then studies were extended to drug loaded Lip-PFP-MSNPs. As already mentioned, a self-designed agar phantom model with circulation tubes was used to simulate and visualize the US contrast. Lip-PFP-Dox-MSNPs were dispersed in deionized water to obtain a concentration of 1 mg/mL MSNPs. After diluting 100 µL of this dispersion in 100 mL of circulating medium, a very good US contrast was observed, even at a very low concentration (1 µg/mL) of MSNPs. The same experiments were performed for lipid coated MSNPs with non-PFP, and they have shown no contrast upon US exposure. The stability of Lip-PFP-Dox-MSNPs was evaluated by contrast characterization for a longer duration. The purpose of this was to observe the retention time and lifespan of liquid PFP in intact form in the pores and its effect on the stability of the carriers at room temperature. For contrast characterization and stability, the particles were kept in the form of purified water dispersion at room temperature. US contrast was observed by adding 100 µL of the sample to the circulating medium at different time intervals of 1 h, 2 h, 24 h, 48 h, 72 h, 96 h, and 120 h. All the samples showed very good US contrast, and even after 120 h the particles were able to show contrast. This confirmed the availability of PFP in the pores as liquid, but upon US-irradiation, the conversion to gas resulted in US contrast, as presented in Figure 4. The results showed that, in the absence of US, PFP containing lipid coated MSNPs are stable for 5 days and can retain the liquid PFP inside their pores. Afterwards, a reduced contrast intensity was observed, which might be due to the loss of PFP.

As shown in Figure 1, a circulating system was used to mimic the bloodstream, where samples showed US contrast in the first circulation; however, in the later circulations, the contrast was reduced, resulting in no contrast at the end. These results support the hypothesis that, upon US exposure, the liquid PFP in the pores vaporizes, leading to loss of PFP in gaseous form by rupturing the liposomal layer and ultimately giving no more contrast. These experiments proved that, at room temperature, the lipid coated MSNPs are stable until they are exposed to US waves. Already reported microbubbles have also demonstrated very good US contrast, but their stability span is limited to a few minutes. In contrast, the developed carriers are very stable for a longer duration and only become unstable when exposed to US-irradiation [47,48].

### 3.4. Ultrasound Triggered Drug Release

**Figure 5:**
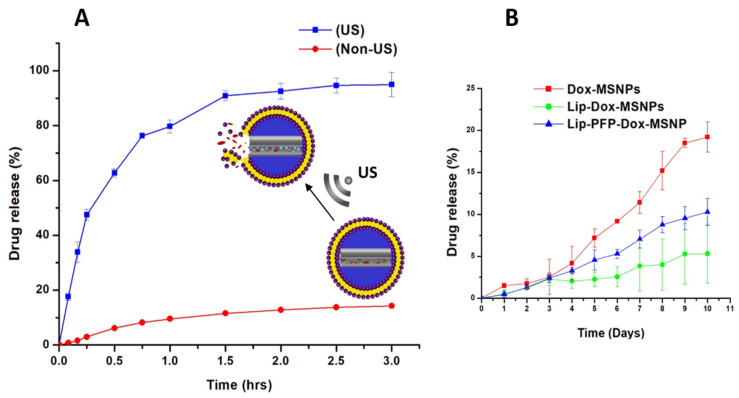
Representative image of (**A**) drug release profile of Doxorubicin from Lip-PFP-Dox-MSNPs *versus* Lip Dox-MSNPs with and without US-irradiation. (**B**) Drug release profile of Dox-MSNPs, Lip-Dox-MSNPs, and Lip-PFP-Dox-MSNPs under inert conditions (Non-US) with PBS buffer at 4 °C.

The Dox loading was calculated with UV-Vis spectrophotometry, as mentioned above, by comparing the Dox concentration in the initial solution used for drug loading to MSNPs. The drug release was also measured in the same way. As per the experimental section, the self-designed agar phantom model was used to assess the drug release. For simulation purpose, PBS at pH 7.4 was used as a medium. To compare the release profile, experiments were performed on Lip-PFP-Dox-MSNPs and Lip-Dox-MSNPs with and without US-irradiation. The probe used had a maximum intensity of 351.37 mW/cm^2^, 12 MHz frequency, and 1.4 MI. According to Levenback et al., at higher intensity, the rise in temperature by US would also be higher. Although there are many factors involved, the absorbance of US by tissues and bubbles is very important and has an inverse relationship with frequency in the case of larger bubbles. On the other hand, smaller bubbles have better absorbance at higher frequencies. Upon US-irradiation, an elevation of temperature up to 50 °C has been observed by many researchers [49]. A lower frequency of 20 KHz can elevate the temperature up to 1000 °C in a matter of microseconds; however, at higher frequencies, it still needs to be evaluated [50]. It was observed that, after US exposure, the Dox had a triggered release and reached its maximum, which was 93% within 3 h, as presented in Figure 5A. However, Lip-Dox-MSNPs, in the absence of US, have shown slower drug release and could reach 10% of the total amount. The experiments on Non-US particles were further extended to evaluate the release behavior, and it was observed that, even after 36 h, the drug release was about 12–13%, which is similar to Xin et al. [51]. Furthermore, the drug release of Lip-Dox-MSNPs (US) and Lip-PFP-Dox-MSNPs (Non-US) at normal body temperature is presented in Supplementary Materials (Figure S3), where it was 19.8% ± 3.46, almost 9% higher than Lip-Dox-MSNPs containing no PFP. This is an indication that such temperature is not enough for vaporization of liquid PFP because of the increased boiling point by the capillary forces in the pores [52].

**Figure 6:**
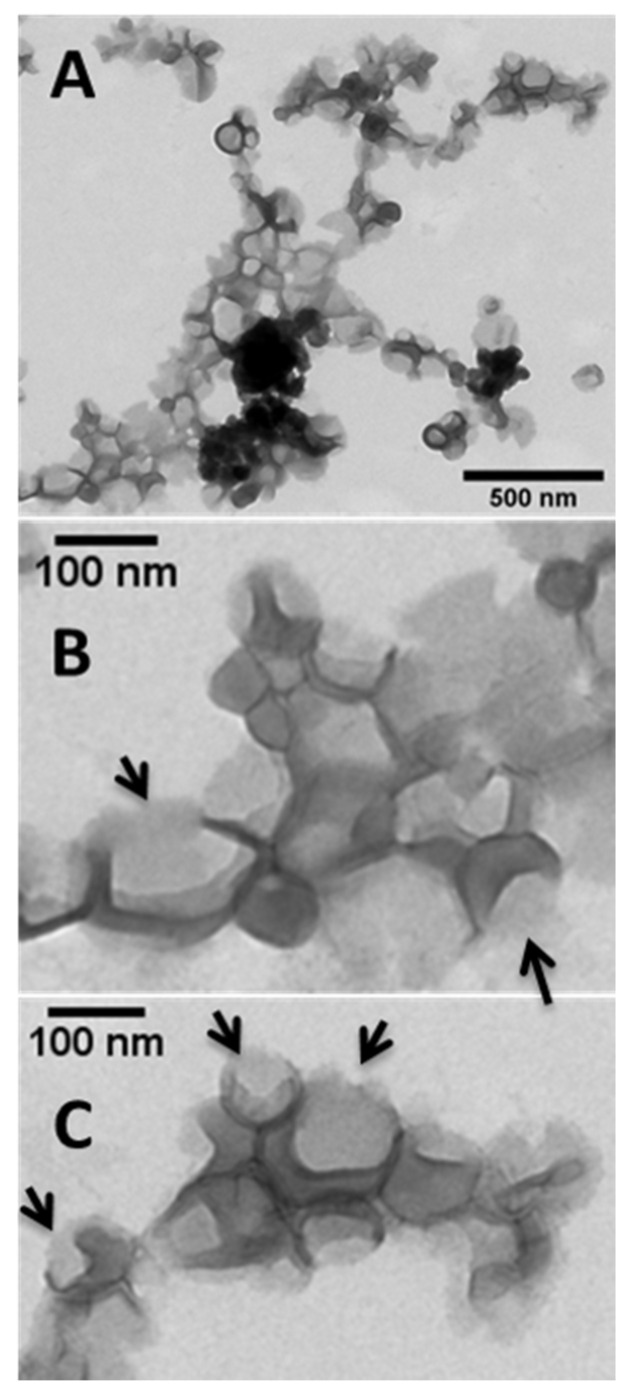
TEM images (**A**–**C**) showing morphology of Lip-PFP-Dox-MSNPs, where the arrows in (**B**,**C**) indicate the areas of lipid bursting after US-irradiation.

The drug release experiments show clear evidence that the introduction of PFP in the pores and particle exposure to US can trigger the release of Dox. A large amount of gas was produced due to vaporization of PFP by US mechanical, thermal, or combined effects. This gas pressure not only ruptured the liposomal coating but also forced the drug release from pores by its mechanical effects. In addition, US thermal induction by acoustic cavitation can also enhance the lipid rupture and provide no more gatekeeping effect [15]. Furthermore, the use of an ultrasound contrasting agent upon US-irradiation can decrease the threshold of cavitation and cavitation related processes [53]. To evaluate the effects of PFP on the lipid layer upon US, the particles were observed under TEM, and images are shown in Figure 6. The lipid layer was negatively stained with 2% uranyl acetate, where bright and dark contrasts represent MSNPs and the lipid coating, respectively. The mesoporous structure of the MSNPs cannot be observed, either because of low magnification or due to structural damage by vaporization upon US. The one side rupture of the lipid layer indicates the exertion of sudden gas pressure, resulting in the lipid opening from its weaker side. In addition to lipid rupturing, the gas pressure was able to damage MSNPs at the edges, causing the triggered release of Dox. Another probability would be the generation of smaller PFP bubbles, their attachment to the lipid coating, and sequentially their disruption by US, leading to rupturing of the lipid layer [15]. By the utilization of US-irradiation in our experiments, and the reported effects of the US reactive agent, a significant difference in drug release was observed, where the triggered release of drugs with US was 9.5 folds higher compared to Non-US.

The role of PFP in Lip-PFP-Dox-MSNPs was analyzed by drug leakage tests and compared with Dox-MSNPs and Lip-Dox-MSNPs. The drug leakage test was helpful for evaluating the effect of PFP on the stability of the drug inside the pores under inert conditions. As presented in Figure 5B, Dox-MSNPs have shown faster Dox release compared to Lip-Dox-MSNPs. The slower Dox release from Lip-Dox-MSNPs is attributed to the presence of the lipid layer as a gatekeeper at the pore openings, and these findings are in accordance with published results where minimum drug leakage was observed from lipid coated MSNPs [40]. On the other hand, Lip-PFP-Dox-MSNPs have shown a slightly faster release of Dox compared to Lip-Dox-MSNPs. This higher release might be due to PFP instability inside the pores when studied for a longer duration, but the rate was still slower than Dox-MSNPs. These findings showed that, under inert conditions, the drug release was not significantly affected by the presence of PFP, but upon US-irradiation, the release was triggered, which is evident in the drug release profile.

As mentioned in the experimental section, a double syringe Luer lock system was used to measure the volume of gas generation by liquid PFP. It was observed that 5 mg of MSNPs contained in 2 mL of Lip-PFP-MSNPs dispersion produced 800 µL of gas (Supplementary Materials, Figure S4) which is a significantly higher amount in comparison to the gas produced in liposomes [38]. Although the volume of entrapped PFP was much lesser, it was still able to produce a relatively large volume of gas. That would have only been possible if PFP was present as liquid and converted to gas, because only liquid can produce such a large volume of gas compared to its original volume. Contrarily, the amount of gas produced would be very low to negligible in the presence of gaseous PFP or in the absence of liquid PFP inside the pores. These findings strengthen the hypothesis regarding the presence of PFP as liquid inside the pores that, upon US-irradiations, was vaporized and ruptured the lipid layer.

### 3.5. Cell Culture Experiments

**Figure 7:**
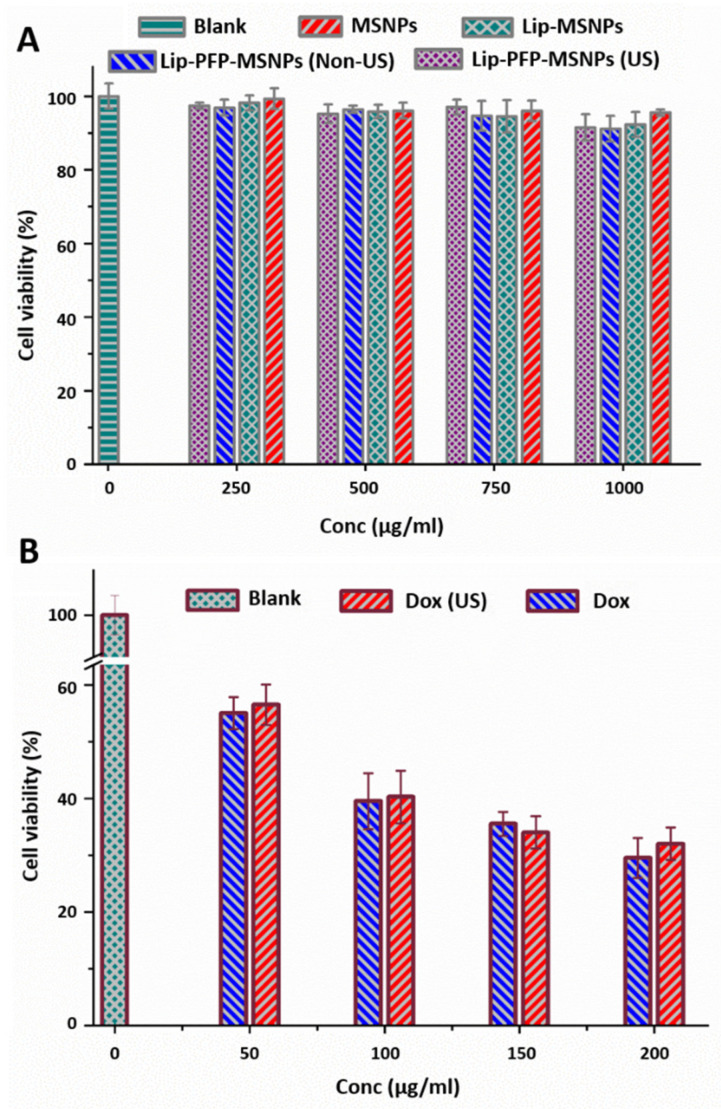
Cytotoxic effects by MTT assay of (**A**) unloaded carriers, including MSNPs, Lip-MSNPs, Lip-PFP-MSNPs (Non-US), and Lip-PFP-MSNPs (US) and (**B**) Dox (Non-US) and Dox (US). US specifications were 5 min, 12 MHz, and MI 1.4, and US-irradiated cells were taken as blank.

The biological compatibility of nanocarriers was evaluated by performing cell viability assay for unloaded particles. MDA-MB-231 cells were seeded in a 5 mL petri dish with DMEM medium supplemented with 10% FCS at a density of 50 × 10^4^ cells/cm^2^ and incubated overnight. Nanocarriers were divided into four different categories, including Lip-PFP-MSNPs (US), Lip-PFP-MSNPs (Non-US), Lip-MSNPs, and MSNPs. In this study, not only the inertness of carriers was evaluated; the effects of PFP and US-irradiation to the cells was also analyzed. All four formulations in different concentrations (250 µg/mL, 500 µg/mL, 750 µg/mL, and 1000 µg/mL of MSNPs) in triplicate were incubated for 4 h, and US-irradiated cells were taken as a blank. Afterwards, medium was replaced, and each plate with PFP-MSNPs (US) was irradiated with US using an eZono device for 5 min. Following overnight incubation, an MTT assay was performed, and it was observed that none of the formulations showed any significant toxic effects. Lip-PFP-MSNPs (US) and Lip-PFP-MSNPs (Non-US) showed the maximum toxicity, which was about 7% at 1000 µg/mL of MSNPs. However, the final concentrations of MSNPs used in the drug-loaded carriers were very low. All the formulations were proven for their safety and biocompatibility and were subjected to further experiments. Similar findings regarding the inertness of nanocarriers have been reported in the literature [24]. The results of biocompatibility are shown in Figure 7A. Before extending our studies to drug-loaded carriers, cytotoxicity experiments with pure drug (US and Non-US) were performed. The sono-sensitivity of the drug was evaluated by the above-mentioned US application, and MTT assay was performed. Here it was observed that, irrespective of US-irradiation, various concentrations of Dox have shown a nonsignificant difference in toxicity, as shown in Figure 7B. Hence, it was confirmed that US-irradiation does not have any major effect on Dox cytotoxicity.

**Figure 8:**
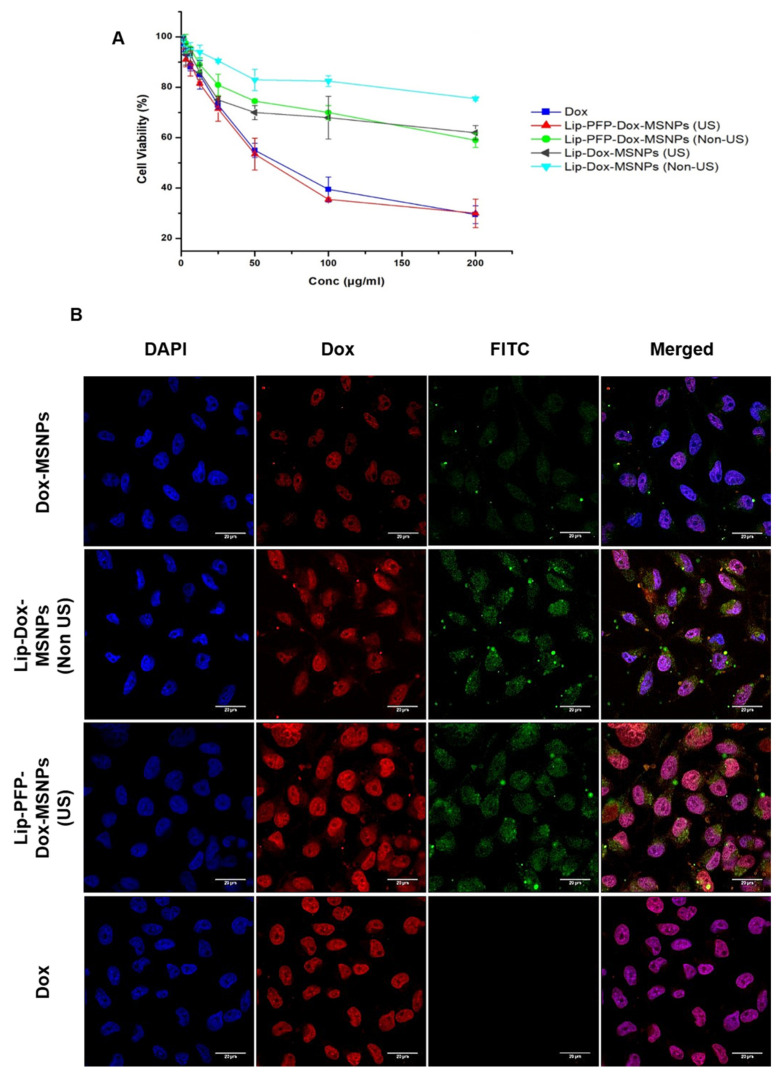
(**A**) In vitro cytotoxic effects of Dox loaded carriers to MDA-MB-231 cells. A comparative cytotoxicity of Lip-Dox-MSNPs and Lip-PFP-Dox-MSNPs with US (5 min, 12 MHz, MI 1.4) and Non-US, where Lip-PFP-Dox-MSNPs (US) showed more cytotoxic effects compared to other groups. (**B**) Cellular uptake studies with confocal microscopy with FITC (green) labelled nanocarriers. Dox-MSNPs, Lip-Dox-MSNPs (Non-US), and Lip-PFP-Dox-MSNPs (US), showing localization of Dox (red) in the nuclei, stained with DAPI (blue), and a scale bar of 20 µm. Higher internalization of Dox with Lip-Dox-MSNPs showed higher cellular uptake due to lipid coating and even higher in Lip-PFP-Dox-MSNPs due to triggered release by US-irradiation, scale bar = 20 μm.

After evaluating carrier inertness and the effects of US on Dox toxicity, the studies were extended to drug loaded carriers. In this section, formulations have been categorized into two groups based on PFP incorporation. In the first group, there was no PFP, and it was further divided into two subgroups (Non-US and US). Similarly, the second group was based on the presence of PFP and consisted of two subgroups (Non-US and US). Cell viability assay was performed for all these formulations by adopting the abovementioned protocol, with Dox as a positive control. After 4 h incubation, US-irradiated samples showed very significant effects. The IC_50_ of Lip-PFP-Dox-MSNPs (US) was 0.105 mM, which is close to pure Dox where its value was 0.108 mM. As per the results presented in Figure 8A, Lip-PFP-Dox-MSNPs (US), in comparison to all other formulations, showed the highest cytotoxicity. The higher cytotoxic effects are in support of the evidence of in vitro US triggered release.

The minimum cytotoxic effects with IC_50_ of 0.417 mM were observed by Lipid-Dox-MSNPs (Non-US), which was due to a lesser release of the drug after 4 h incubation. Although the lipid coating enhanced the cellular uptake, the carriers were still not able to release complete Dox. On the other hand, Lip-PFP-Dox-MSNPs (Non-US) and Lip-Dox-MSNPs (US) with IC_50_ of 0.222 mM and 0.236 mM, respectively, have shown relatively higher cytotoxic effects, but they are similar to each other. In comparison to the formulation without PFP and US, the faster release from Lip-PFP-Dox-MSNPs (Non-US) and Lip-Dox-MSNPs (US) was due to the presence of PFP and US-irradiation, respectively. It can be assumed that evaporation of a very small amount of PFP in Lip-PFP-Dox-MSNPs resulted in drug release, even in the absence of US, but this release was higher than the release from Lip-Dox-MSNPs and ultimately produced more cytotoxic effects. Similarly, higher cytotoxic effects were shown by Lip-Dox-MSNPs (US) compared to Lipid-Dox-MSNPs (Non-US), which is due to US-irradiation.

Although the reason behind these results is unknown, it can be assumed that the higher release was related to some vibrational movements imparted by the mechanical energy of US on the porous structure. The cytotoxic effects produced by Lip-PFP-Dox-MSNPs (Non-US) and Lip-Dox-MSNPs (US) were lesser than Lip-PFP-Dox-MSNPs (US) because, in the later, PFP was incorporated and vaporized by the thermal and mechanical effects of US. The FDA approved US waves for clinical purposes, with MI 1.4 and a frequency of >10 MHz can penetrate to the surface tissues [8,54], and in cell culture experiments after cellular uptake of nanocarriers, US-irradiation has produced significant results. The PFP inside the pores of carrier, being prone to be disturbed by US-irradiation, can trigger the release of the drug inside the cells [14]. Consequently, Lip-PFP-Dox-MSNPs (US), compared to all three formulations, have shown higher cytotoxic effects. Hence, it has been revealed that the combined effect of PFP presence in MSNPs and US-irradiation was able to trigger the intracellular drug release and enhanced the cytotoxic effects in in vitro tumor cells.

### 3.6. Intracellular Drug Release and Cellular Uptake Studies

The intracellular triggered release of the drug was further evaluated by cellular uptake studies, where, after internalization of the carriers, cells were observed under fluorescence microscope, and the results are presented in Figure 8B. Lipid coated MSNPs, due to higher biocompatibility, enhanced the cellular uptake and drug delivery, which has already been reported by researchers [42,55]. Three different formulations, including Lip-PFP-Dox-MSNPs (US), Lip-Dox-MSNPs, and Dox-MSNPs were used for internalization and cellular uptake studies. To avoid the toxic effects, lower concentrations of Dox were used for 4 h incubation. After US-irradiation and fixation, nuclei stained cells were observed under CLSM. Green fluorescence is characteristic of FITC labelled MSNPs, while red fluorescence is characteristic of Dox. As seen in Figure 8B, the cytoplasmic region with green fluorescence confirmed the efficient uptake and localization of nanocarriers. Lip-PFP-Dox-MSNPs (US) and Lip-Dox-MSNPs (Non-US) were highly internalized compared to Dox-MSNPs, which is attributed to lipid coating to enhance the cellular uptake [56]. The cellular localization of Dox also showed the uptake pattern, as mentioned above. A lower fluorescence of Dox in Dox-MSNPs compared to lipid coated formulations was due to lower cellular uptake of the carriers. On the other hand, a significantly higher Dox localization in Lip-PFP-Dox-MSNPs was observed, which was due to enhanced drug release with US-irradiation. The intracellular release pattern is in accordance to in vitro release studies where the release was triggered by US-irradiation.

## 4. Conclusions

This study involved the successful development of a stimuli responsive drug delivery system where Dox was used as a model drug, PFP as a stimuli responsive agent, US as a stimulus, and lipid coated MSNPs as carriers. The combined effects of liposomes and MSNPs could produce very significant results due to the biocompatibility and gatekeeping effects of the liposomes, along with the unique structure and morphology of MSNPs. Highly stable nanocarriers were produced by incorporation of PFP inside the pores of MSNPs via capillary filling. The FDA approved US specifications were utilized to produce satisfactory triggered release effects by rupturing the lipid layer due to vaporized PFP pressure. In vitro experiments with breast cancer cells showed that nanocarriers were highly internalized due to the biocompatible nature of the lipid layer. Intracellular US triggered release produced higher cytotoxic effects. These smart nanocarriers can be utilized to incorporate and deliver various chemotherapeutics for triggered release at specific sites and to avoid unwanted effects with enhanced efficiency.

## Figures and Tables

**Table 1:** Particle size, polydispersibility index (PDI), and zeta potential of MSNPs, liposomes and Lip-MSNPs by DLS.

	Particle Size ± SD (nm)	PDI	Zeta Potential ± SD (mV)	
MSNPs	118 ± 20	0.159	+34 ± 5	Before washing
			−17 ± 4	After washing
Liposomes	126 ± 12	0.177	+18 ± 4	
Lip-MSNPs	130 ± 10	0.242	+8 ± 3	

## Data Availability

Not applicable.

## Supplementary Materials

The following are available online at https://www.mdpi.com/article/10.3390/pharmaceutics13091396/s1, Figure S1. Atomic force microscopic images from different areas and magnifications where MSNPs are shown in amplitude trace (I), measured height trace (II) along with graphs (a–f) of different particles (III) representing the size distribution. Figure S2. Surface area and pore size evaluation by (A) nitrogen adsorption-desorption and according to BET method the surface area of MSNPs has been increased from 8.3 m^2^/g to 1054 m^2^/g after surfactant removal, (B) BJH pore diameter is 2.43 nm. Figure S3. Drug release profile of Lip-PFP-Dox-MSNPs and Lip-Dox-MSNPs with and without US at 37 °C, showing the triggered release from Lip-PFP-Dox-MSNPs just upon US irradiation. Figure S4. Two way Luer lock system used for the measurement of gas produced where (A) is showing the volume before US, while (B & C) showing the volume of gas produced after US irradiation.
