# Supplementary material for: Ultrasound-Responsive Smart Drug Delivery System of Lipid Coated Mesoporous Silica Nanoparticles
Source: Pharmaceutics. 2021 Sep 3;13(9):1396. doi: 10.3390/pharmaceutics13091396 (PMC8468042; doi:10.3390/pharmaceutics13091396)
Supplement: Supplementary file 1 [file pharmaceutics-13-01396-s001.zip › pharmaceutics-1326281-supplementary.pdf]

pharmaceutics

MDPI

Supplementary Materials: Ultrasound-Responsive Smart Drug Delivery System of Lipid Coated Mesoporous Silica Nanoparticles

Muhammad Umair Amin, Sajid Ali, Imran Tariq, Muhammad Yasir Ali, Shashank Reddy Pinnapreddy, Eduard Preis, Christian Wölk, Richard D. Harvey, Gerd Hause, Jana Brüßler and Udo Bakowsky

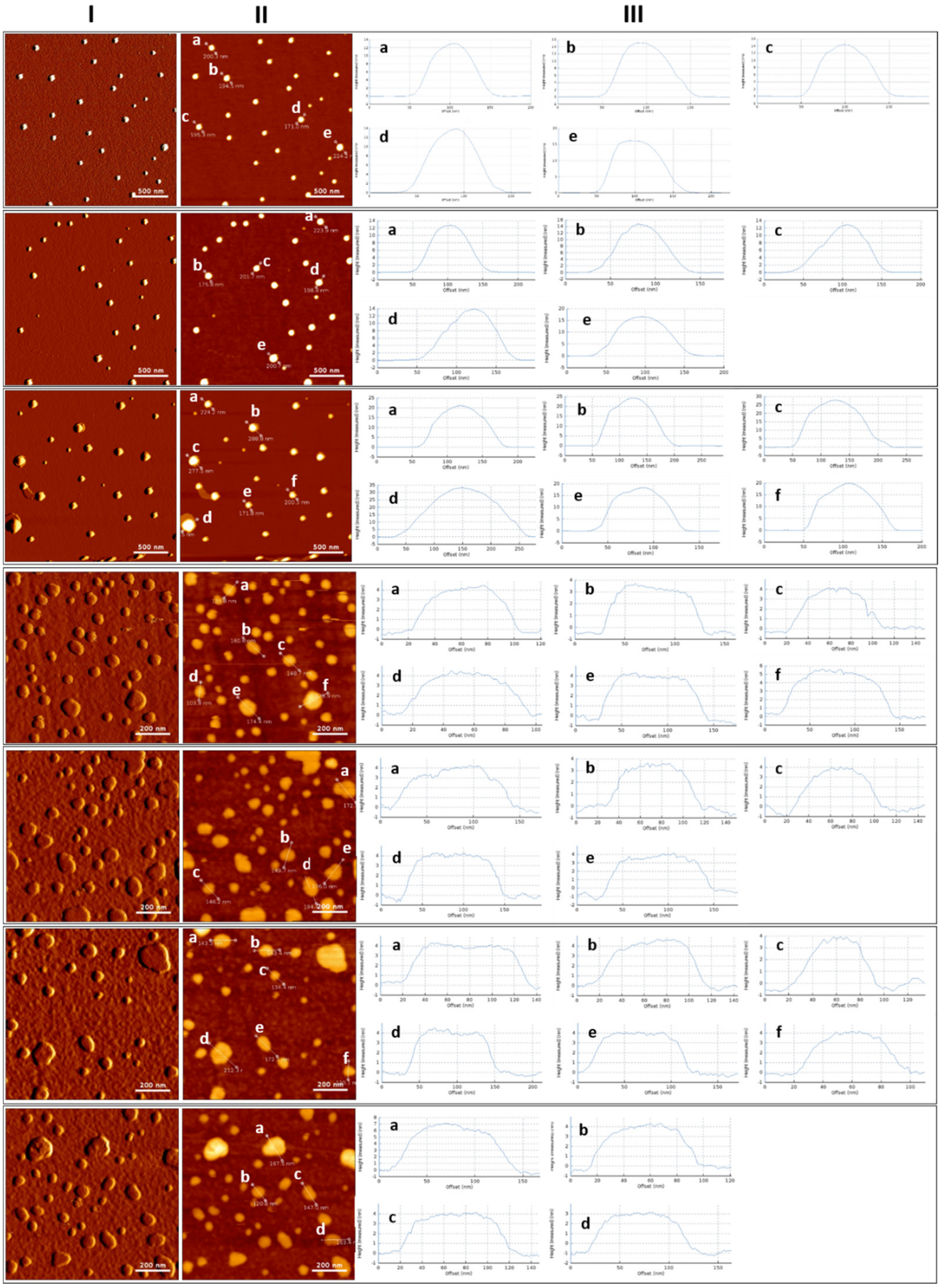

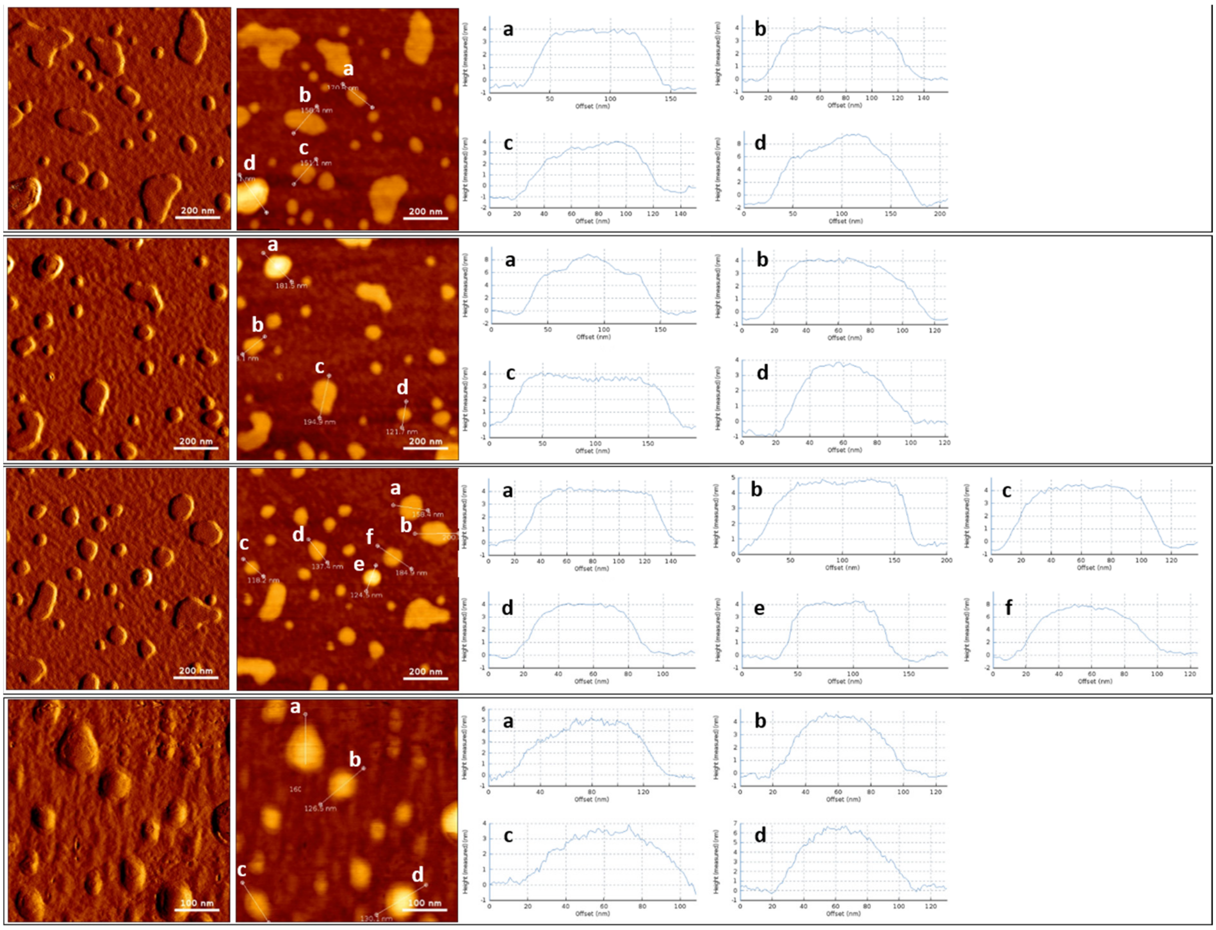

Figure S1. Atomic force microscopic images from different areas and magnifications where MSNPs are shown in amplitude trace (I), measured height trace (II) along with graphs (a-f) of different particles (III) representing the size distribution.

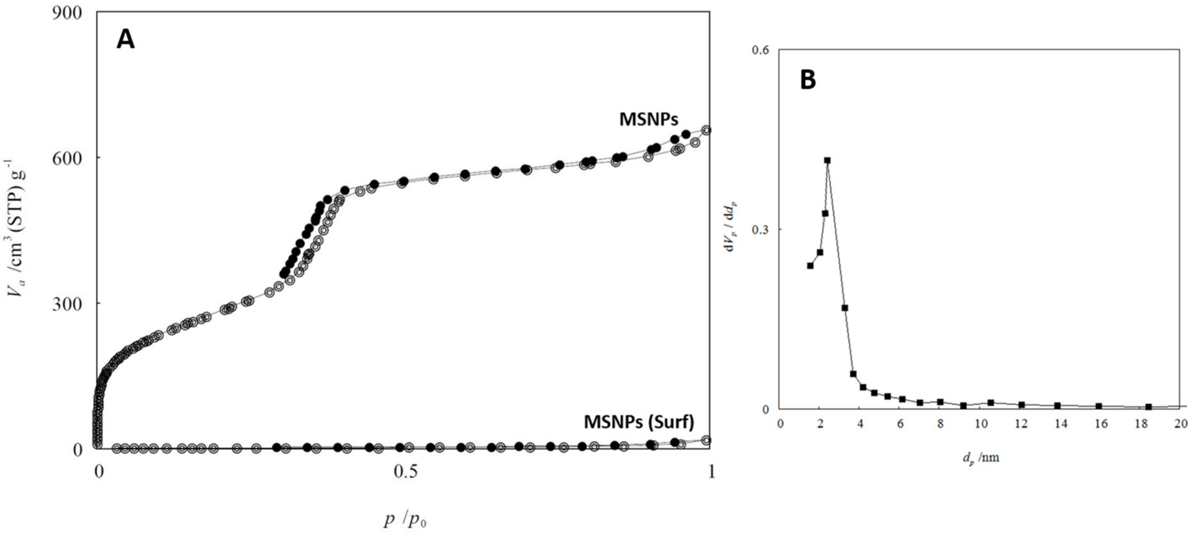

Figure S2. Surface area and pore size evaluation by (A) nitrogen adsorption-desorption and according to BET method the surafce area of MSNPs has been increased from 8.3 m^2/g to 1054 m^2/g after surfactant removal, (B) BJH pore diameter is 2.43 nm.

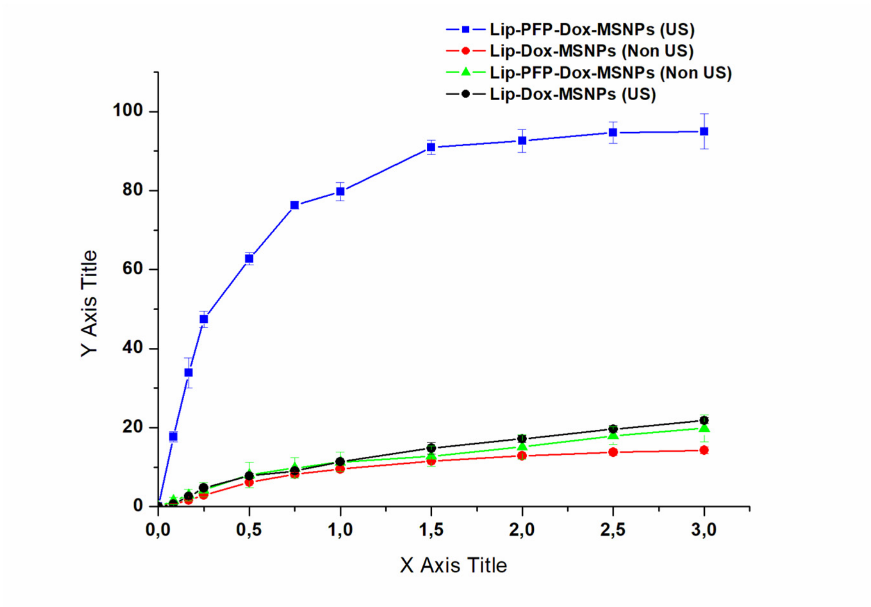

Figure S3. Drug release profile of Lip-PFP-Dox-MSNPs and Lip-Dox-MSNPs with and without US at 37°C, showing the triggered release from Lip-PFP-Dox-MSNPs just upon US irradiation.

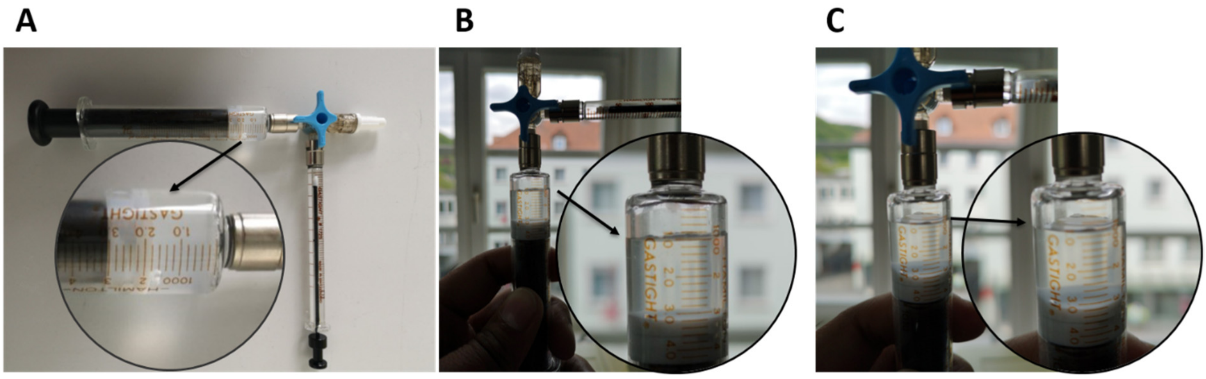

Figure S4. Two way Luer lock system used for the measurement of gas produced where (A) is showing the volume before US, while (B & C) showing the volume of gas produced after US irradiation. The circles are magnified areas indicated with black arrows.
